# Density dependent habitat selection in response to habitat loss in a coral reef fish

**DOI:** 10.1111/1365-2656.70135

**Published:** 2025-09-19

**Authors:** Lisa Boström‐Einarsson, Mary C. Bonin, Philip L. Munday, Geoffrey P. Jones, Sally A. Keith

**Affiliations:** ^1^ Lancaster Environment Centre Lancaster University Lancaster, Bailrigg UK; ^2^ ARC Centre of Excellence for Coral Reef Studies James Cook University Townsville Queensland Australia; ^3^ Great Barrier Reef Foundation Brisbane City Queensland Australia; ^4^ College of Science and Engineering James Cook University Townsville Queensland Australia

**Keywords:** coral reef fishes, density‐dependent habitat selection, ecological traps, habitat degradation, habitat loss, *Pomacentrus moluccensis*

## Abstract

1. Habitat degradation alters the availability and quality of resources, with ramifications for how populations distribute across remnant patches. Decisions about habitat use are often made to optimise fitness by minimising competition for resources. Individuals can sort themselves optimally across patches by selecting habitat based on the density of resident individuals, yet it is unclear whether this mechanism is disrupted by habitat loss. Moreover, density‐dependent habitat selection could create a socially reinforced ‘bandwagon’ effect in species that use adults as a positive settlement cue.
2. Here, we aimed to (1) determine the effect of habitat degradation on density‐dependent habitat selection and (2) test whether habitat use by adults influences settlement decisions by juveniles via a bandwagon effect in the coral‐associated reef fish, *Pomacentrus moluccensis*.
3. We combined field surveys with a habitat choice experiment in Pomacentrus moluccensis to assess how fish respond to varying coral quality and conspecfici densities.
4. Field observations revealed that adults only used dead coral on degraded reefs where fish densities on surrounding remnant live colonies were exceptionally high. When presented experimentally with the choice of two colonies, fish were more likely to choose a near empty alternate colony when the other colony was severely crowded with conspecifics.
5. Taken together, these results offer strong support for density‐dependent habitat selection during habitat loss. This choice cascades to influence juvenile habitat use: juveniles selected dead corals to a greater extent if there was a conspecific adult present. To our knowledge, this is the first empirical demonstration of how habitat degradation can trigger density‐dependent habitat selection, which in turn may shape settlement decisions in the next generation via socially mediated cues.

## Graphical abstract

Habitat loss triggers a social chain reaction: adult reef fish crowd onto remaining coral, then spill over onto dead coral—and juveniles follow. This study reveals a novel, socially driven ‘bandwagon effect’ that may lead to ecological traps, highlighting hidden behavioural risks in degraded marine ecosystems.

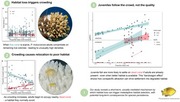

## 1 INTRODUCTION

Resource quality and availability are critical determinants of species distribution and abundance (Fretwell, 1972; Fretwell & Lucas, 1969). While the direct effects of environmental disturbance on resources are increasingly well documented, the indirect effects of disturbance in mediating access to resources are poorly understood. Individuals often select suitable habitat of high resource quality based on cues related to existing users of that resource. However, the more individuals that access a finite resource, the less valuable it becomes per capita. Furthermore, in species with a high level of conspecific attraction, habitat selectivity by one subset of individuals or life stages can influence decisions by subsequent arrivals creating a ‘bandwagon effect’ (Swartwout, 2007). Ultimately, habitat selection and resource use largely determine the distribution of species in heterogenous landscapes and rely on the capacity of species to respond appropriately to cues in their environment. Our knowledge of how these processes play out during disturbance events is unresolved, while the indirect impact on subsequent arrivals is completely unknown.

When resources are limited, which is commonly a result of disturbance, individuals may choose less crowded but lower quality habitat patches to avoid the negative impact of competition (Macarthur & Levins, 1964). As more individuals access a certain habitat patch, the relative value of the patch to each individual declines. This process, where individuals distribute themselves among habitats of varying quality such that their expected fitness payoffs are equalised, is known as density‐dependent habitat selection (Morris, 1989; Rosenzweig, 1981). Models such as the Ideal Free Distribution (Pulliam & Danielson, 1991) and Shima and Osenberg's cryptic density‐dependent framework (Osenberg et al., 2006; Shima & Osenberg, 2003) predict this pattern by considering how crowding and site quality jointly influence habitat selection and subsequent fitness. The latter model focuses specifically on habitat selection at the time of larval settlement, assuming that individuals assess both habitat quality and conspecific density in making fitness‐optimising choices. While these models provide a framework for understanding habitat selection, their role in determining responses to habitat degradation, particularly in the context of post‐settlement habitat shifts, has never been empirically tested. Habitat selection often relies on a combination of direct and indirect cues. Direct cues may include physical structure, olfactory signals or visual properties of the habitat itself. Indirect cues, such as the presence of conspecifics, can serve as proxies for habitat quality (Stamps & Krishnan, 2005), particularly in species or life stages where assessing habitat directly is difficult. In many species, juveniles use the presence of adult conspecifics as an indirect cue to infer high quality habitat (Buxton et al., 2020), although the extent to which this occurs varies across taxa. However, if juveniles follow adults to lower quality habitats based on incorrectly interpreted cues, it could create a bandwagon effect, a term borrowed from the social sciences (Swartwout, 2007). Here, an increased prevalence of a behaviour at the population level increases the probability of that behaviour at the individual level (Boto‐García & Baños‐Pino, 2022). In such a scenario, the consequences for affected populations will ultimately depend on whether this behavioural change increases or decreases fitness of affected individuals. While bandwagon effects have been described in the context of consumer behaviour (Leibenstein, 1950), voting behaviour (Farjam, 2021), technology adaptation (Rohlfs, 2003) and the opinion formation process (Nadeau et al., 1993), the concept remains unexplored in ecology. However, given that dynamic ecosystems undergoing rapid environmental change (e.g. coral reefs) are primed to see this chain of events unfold, it is critical to improve our understanding of the link between habitat loss, density‐dependent habitat selection and any resulting bandwagon effects.

Coral reefs offer an excellent model system with which to explore these concepts because they experience increasingly severe and frequent disturbance (Hughes et al., 2021; Souter et al., 2020), acting as an early indicator in the unfolding climate crisis (Sweet et al., 2021). Corals offer an easily quantifiable resource—many fish species are reliant on live corals at some stage in their life cycle, either during recruitment as larvae (Bonin, 2012; Coker et al., 2013; Jones et al., 2004), as a food source (Cole et al., 2008; Pratchett et al., 2004) or to seek refuge from predators (Boström‐Einarsson et al., 2018; Holbrook & Schmitt, 2002). Consequently, there are well‐documented fitness benefits of access to live corals (Pratchett et al., 2018), where coral reef fishes on degraded or dead coral colonies exhibit lower survival (Boström‐Einarsson et al., 2018; Noonan et al., 2012), growth (Feary et al., 2009; Kokita & Nakazono, 2001), reproductive potential (Brooker et al., 2013) and physiological condition (Pratchett et al., 2004). The exact mechanisms that lead to dramatic reductions in abundance of reef‐associated fishes are often unknown, but likely culprits are increased competition for resources (Bonin et al., 2015), the long‐term outcomes of sub‐lethal fitness effects (Booth & Beretta, 2004; Hoey & McCormick, 2004) and altered behaviour (Boström‐Einarsson et al., 2018).

Small‐bodied reef fishes use a combination of senses, including vision, smell and hearing, to identify suitable habitat on the reef (Atema et al., 2002; Lecchini et al., 2005; Majoris et al., 2021; Tolimieri et al., 2000). Juvenile reef fish use these highly attuned senses to judge habitat quality at or near the time of settlement. While many studies focus on cue use by pelagic larvae during initial settlement, conspecific attraction may also shape habitat choices by recently settled individuals, for example, through post‐settlement movement between nearby corals (Booth, 1995; Sweatman, 1983; Ward et al., 2020). The vulnerability of coral reef ecosystems to habitat loss, and reef fishes' high reliance on live corals, makes them excellent study systems for exploring behavioural responses to habitat loss.

Here, we test how resource (live coral) decline affects habitat selection in a common coral‐associated reef fish species, *Pomacentrus moluccensis*, using observations and experiments in the field. We use a field‐based study to (1) quantify habitat use of adult and juvenile *P. moluccensis* on reefs with a range of disturbance levels following a large‐scale degradation event and test whether habitat degradation has created a bandwagon effect for these abundant reef fishes. Specifically, we (2) test whether the presence of adults on a coral colony can predict juvenile habitat use and (3) assess the sustained density effects of the degradation event on this fish community after 12 months. Finally, we (4) use a choice experiment to quantify how habitat selection in juvenile *P. moluccensis* is influenced by both increasing conspecific density and the quality of the alternative habitat.

## 2 MATERIALS AND METHODS

### 2.1 Study site and species

The study was conducted on shallow platform reefs in Kimbe Bay, Papua New Guinea (150°05′ E, 5°25′ S), in April 2013 during an outbreak of crown‐of‐thorns starfish (COTS, *Acanthaster* cf. *solaris*). The outbreak was characteristically patchy in nature, with some reefs severely affected while others were only mildly affected, creating a gradient of disturbance that we used to explore the influence of habitat degradation on distribution patterns and habitat use by the common coral‐associated reef fish *P. moluccensis*. Our surveys capture a spatial gradient in disturbance severity, and our inferences about behavioural responses to habitat loss are based on a space‐for‐time substitution framework (Damgaard, 2019; Pickett, 1989). While some reefs were actively degrading due to COTS outbreaks at the time of the study, our data represent a spatial snapshot rather than a temporal trajectory.

### 2.2 Distribution and habitat use of *P. moluccensis*

We recorded the local distribution of *P. moluccensis* across its primary habitat using 50 × 2 m belt‐transects (*n* = 49) along the exposed reef crest of 12 shallow platform reefs. The start of each transect was marked using a handheld GPS, and transects were separated from each other by a minimum of 5 m. Within each 100‐m^2^ transect, all plating and corymbose *Acropora* colonies were surveyed and scored into four categories based on the presence of *P. moluccensis* (occupied/unoccupied) and whether the colony was alive or dead. Corals with >80% dead coral areas were scored as ‘dead’ while remaining colonies were scored as live. For occupied colonies, the number of resident *P. moluccensis* was counted and recorded as adults or recent settlers (juveniles identified based on size, <10 mm and pale body colouration). Planar images of each colony were then taken (with ruler for scale), and the surface area of each colony was measured in ImageJ (Schneider et al., 2012). Together with the fish count, these were used to calculate the density of fish at each colony.

We modelled the effect of local habitat degradation on the density of *P. moluccensis* on coral colonies using a linear regression. We used the density of *P. moluccensis* on each occupied coral colony as a response variable, and the proportion of dead coral at the transect level and the coral colony type (live/dead), as well as the interaction between the two, as explanatory variables. We used transect level data as our explanatory variable under the assumption that *P. moluccensis* habitat selection is likely to be dictated by processes at this local scale. We checked assumptions visually and subsequently log‐transformed the response variable. To test for sudden switches in the modelled relationship, we used change point analysis on the live coral data only using the R package mcp (Lindeløv, 2020). Based on visual evaluations of the data, we modelled a single change point in variability.

### 2.3 Density‐dependent habitat selection choice experiment

To experimentally test for density‐dependent habitat selection, we performed a habitat choice experiment in underwater enclosures. *P. moluccensis* juveniles were allowed to choose between a 100% live coral colony with an established group of conspecifics (13–59 individuals) and an alternate colony (either live or dead) that had a very low density of conspecifics (two individuals). This design ensured that choices made by juveniles were strictly related to the density of conspecifics of the established colony and the quality of the alternate colony and not confounded by avoidance of empty colonies by this highly aggregative species. The coral colonies used in the experiment were all corymbose *Acropora* colonies of a similar size (~30 cm diameter). We used scaled images in imageJ to measure the colony surface area for fish density calculations. We used five density levels on the established colony (~142, 165, 186, 326 and 663 fish m^−2^) ranging from those normally encountered on healthy reefs to extreme levels that were above those normally experienced in the reef environment. Using levels that exceed those found in nature allowed us to effectively measure and detect density‐dependent effects (Forrester et al., 2006; Inouye, 2001). Fish were restricted to the experimental enclosure but were able to move freely between the two coral colonies within it. The experimental cages were positioned on an isolated patch of sand approximately 10–15 m away from any substantial reef or coral formations. This placement was chosen deliberately to minimise external visual or olfactory cues and to ensure that focal fish interacted primarily with the experimental coral colonies and conspecifics within the enclosure.

**FIGURE 1:**
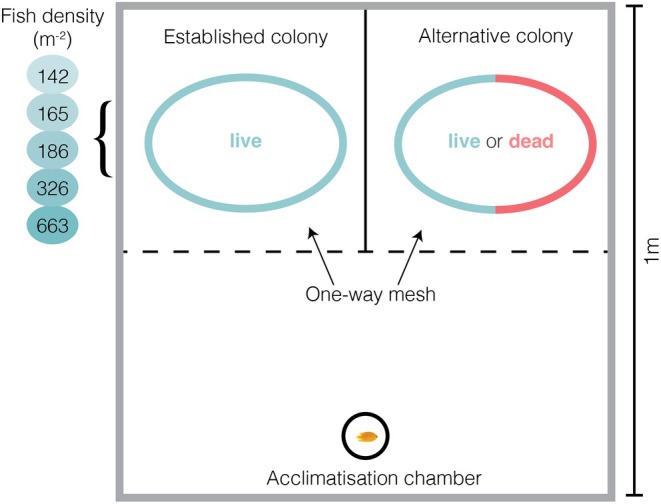
Choice experiment arena testing whether habitat preferences of settling *P. moluccensis* juveniles are influenced by increasing conspecific densities. The established colony consisted of a live corymbose *Acropora* coral colony, stocked with five density levels of *P. moluccensis* (between 142 and 663 per m^2^). The alternative colony consisted of either live or dead corymbose *Acropora* colony stocked with two *P. moluccensis*. The mesh allowed juvenile *P. moluccensis* to pass through but prohibited the larger adults from leaving their colonies.

Recent settlers were identified based on their small size (<10 mm) and pale body colouration, both of which are characteristic of *P. moluccensis* within the first 24 h post‐settlement. Individuals at this stage have not yet developed the bright yellow colouration typical of older juveniles. Surveys were conducted at first light to maximise the likelihood of detecting these early post‐settlement individuals before rapid colour changes occurred. These individuals had settled the previous night, meaning that our experiment tested post‐settlement movement and fine‐scale habitat selection, rather than initial larval settlement. However, prior work on *P. moluccensis* (Öhman et al., 1998) has shown that habitat preferences expressed immediately post‐settlement are consistent with those of naïve larvae, suggesting that the patterns we observed are a reliable proxy for habitat preferences at settlement. Newly settled juveniles were collected using dilute clove oil and hand nets and placed in clip seal bags. Fish used in the established colonies were collected from existing groups so that social dynamics and hierarchies were already established. Four replicate cages were placed in a shallow sandy area (approximately 3 m depths). Cages were 1 m × 1 m squares, with internal dividers (Figure 1). External walls, roof and colony divider were constructed of wire and plastic mesh (mesh size <5 mm) so that water could pass through yet prohibiting movement of fish. The cages contained three chambers; the main chamber and two colony chambers containing the established and alternate colonies.

Juveniles were placed in an acclimatisation tube (PVC pipe 75 mm diameter with mesh lid) in the main chamber for 30 min, after which the chamber was removed by pulling a 2‐m‐long string (to reduce disturbance to the fish). Each choice trial was started between 16:00 and 17:00, allowing the juvenile to make a choice overnight. The chosen colony was recorded the following morning by visually inspecting the cages between 7:00 and 8:00 AM. Choice tests were replicated 10 times at each of the five density levels for both alternate colony types (i.e. live or dead), resulting in 100 total tests. A new fish was used in each choice trial to ensure that individuals were naïve to the experimental system and choices were not affected by prior learning. Coral colonies used in the trials were from the same species and growth morphologies as those commonly used by *P. moluccensis*; however, we did not match the coral species from where fish were collected to the trial colonies.

The effect of habitat quality and conspecific density of the established colony on the habitat choice of a juvenile *P. moluccensis* was compared using a logistic regression. The model predicted the colony choice of a *P. moluccensis* juvenile (established colony = 0, alternate colony = 1) as a function of the density of resident *P. moluccensis* on the established colony and the quality/condition of the alternate colony (live or dead). The fit of the selected model was evaluated using a likelihood ratio test, and the significance of each term in the reduced model was evaluated using a Wald chi‐squared test.

### 2.4 Does the presence of adults on a coral colony predict juvenile habitat use?

To explore whether adult habitat use influences juvenile habitat selection, a key feature of the proposed ‘bandwagon effect’, we used Manly's selection ratios (design III; Calenge, 2006) to assess juvenile habitat preference at the transect level. This method compares the proportion of each habitat type available (live vs. dead coral colonies) to the proportion actually used by juveniles (i.e. occupied colonies), providing a measure of selection or avoidance. A value greater than 1 indicates preference, while values less than 1 suggest avoidance. We calculated these ratios for each transect and modelled them using linear regression to test whether juvenile habitat selection varied with the proportion of dead coral on the reef.

### 2.5 Effects of habitat loss on *P. moluccensis* abundance

To compare how the overall abundance of *P. moluccensis* was affected by the crown‐of‐thorns outbreak, we then conducted repeat surveys of fish abundances at the transect level after 12 months at the same locations. We compared the mean overall abundance of *P. moluccensis* at the start of the study in April 2013 (early outbreak) to April 2014 (12 months later) using a Student's *t*‐test.

All research was conducted under James Cook University animal ethics approval No: A1899.

## 3 RESULTS

### 3.1 Distribution and habitat use of *P. moluccensis*

**FIGURE 2:**
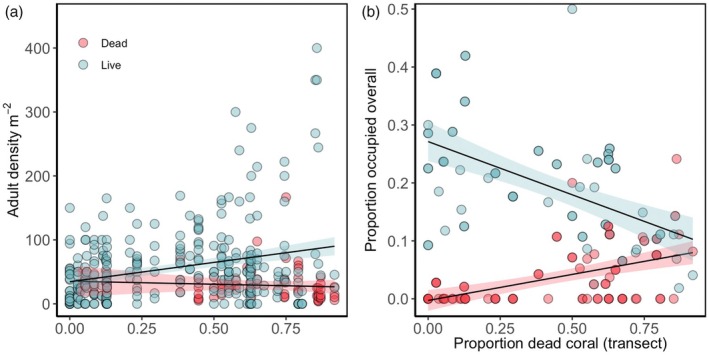
(a) The density of adult *P. moluccensis* aggregations on coral colonies at transects with a varying proportion of dead coral colonies. Each data point represents a single coral colony (*n* = 379), across transects (*n* = 49). (b) The proportion of coral colonies occupied by at least one adult *P. moluccensis*. Each transect is represented by one data point per coral type (live or dead), providing each type is present (*n* live = 48, *n* dead = 23).

We surveyed the habitat use of *P. moluccensis* on 379 coral colonies across 49 transects ranging from 0 to 91% dead coral. Habitat use by *P. moluccensis* varied depending on the amount of dead coral in the immediate vicinity. There was a significant positive relationship between the density of adult *P. moluccensis* on coral colonies and the proportion of dead coral on a transect, and the slope of this relationship differs depending on whether the coral colony is live or dead (Figure 2a, two‐way ANOVA, interaction term *t* = 2.5, df_(3375)_, *p* = 0.01). There is more than a twofold increase in the average density of *P. moluccensis* per live colony on transects with <25% compared to those with >75% dead coral (mean density 53.8 ± 3.5 SEM and 121.4 ± 24.1, respectively) as well as a substantial increase in variability. Indeed, the change point analysis indicated a shift in variability when dead coral exceeded approximately 50% on the transects (Figure S1). Importantly, there are remnant live coral colonies available on each transect regardless of the degree of disturbance experienced (Figure 2b).

When less than 50% of coral colonies on a transect were dead, *P. moluccensis* very rarely occupied dead colonies (Figure 2b). In contrast, above this threshold, *P. moluccensis* started using a small proportion of dead habitat. When more than three quarters of coral colonies were dead, *P. moluccensis* occupied an equal number of dead and live coral colonies.

### 3.2 Density‐dependent habitat selection choice experiment

**FIGURE 3:**
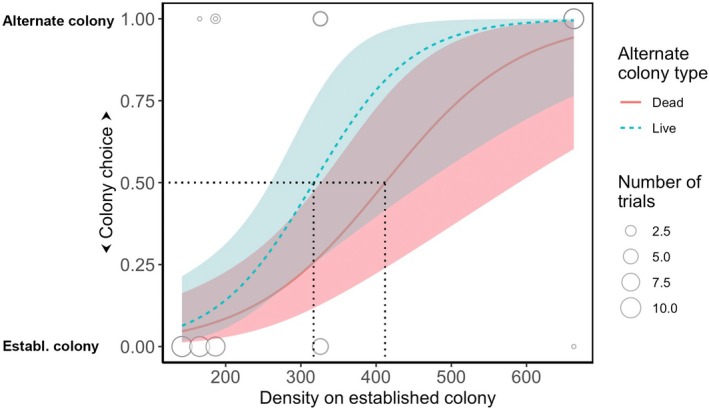
Logistic regression of colony choice of juvenile *P. moluccensis*. Established colony (colony choice = 0) was occupied by an aggregation of *P. moluccensis* adults and sub‐adults in densities ranging from 142 to 663 individuals m^−2^ (*x*‐axis). The alternate colony (colony choice = 1) was occupied by two adult *P. moluccensis* and was either live or dead. Dotted lines indicate that the densities above which 50% of the juveniles are likely to choose the alternate colony. Size of point indicates number of trials with that outcome. Shaded bands indicate 95% confidence intervals around model prediction.

**FIGURE 4:**
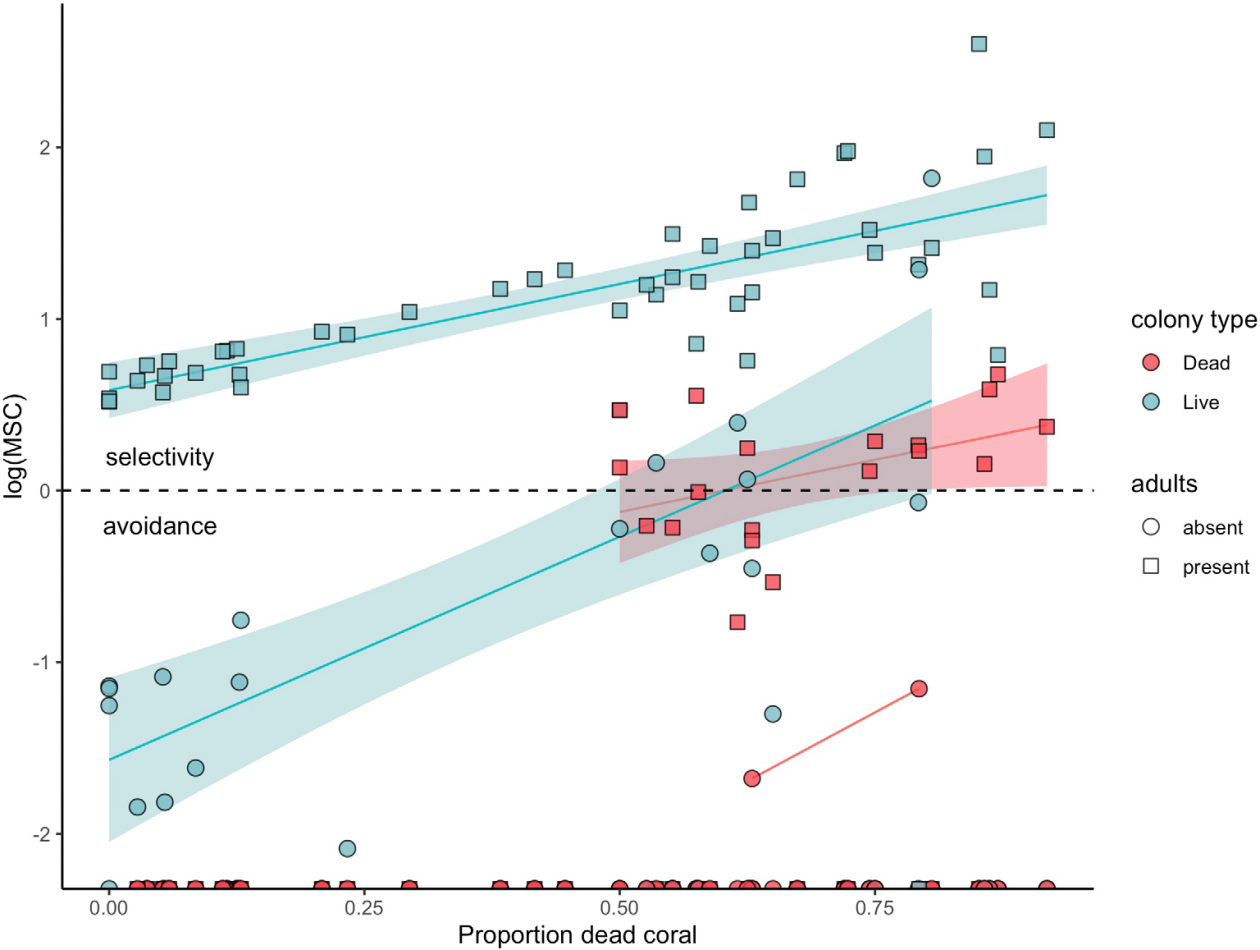
Manly's selection criteria (MSC) at transect levels for four categories of coral colonies with *P. moluccensis* juveniles (dead/live; adults present/absent). A selection ratio >0 (note log scale) indicates that *P. moluccensis* is using that habitat category to a greater extent than what would be expected based on its availability.

The density of adults on the established coral colony had a significant effect on the habitat choice of *P. moluccensis* juveniles in the habitat selection experiment (Table 1). Juveniles were more likely to choose the alternate colony when the density of adult residents was high (Figure 4). There was no significant difference in choice between the live and dead coral colony with a low‐density conspecific cue (Table 1), although there was a suggestion that fish were more reluctant to choose the dead than the live coral colony (Figure 3). For example, when the alternative colony was live, the model predicts that more than 50% of the fish will choose it over the established colony if densities are above 317 ± 70.5 (95% CI) fish m^−2^. If the alternative colony is dead; however, the equivalent density on the established colony was 412 ± 103 fish m^−2^.

**TABLE 1:** Logistic regression of colony choice by juvenile *P. moluccensis*. Fish were allowed to choose between a colony with an established group of conspecifics (five density levels of aggregation) and an alternate colony (live or dead) which had a very low density of conspecifics.

Factors	*β*	SE *β*	*χ* ^2^	df	*p*
Constant	−5.07	0.97			
Density (est. colony)	0.01	0.003	20.7	1	<0.0001
Alt. colony type	−0.87	0.69	1.6	1	0.2
Likelihood ratio test (full model)			62.7	2	<0.0001

*Note*: The established colony was stocked with a range of densities of *P. moluccensis*, while the alternate colony type was either live (dummy coded 1) or dead (2). The Wald chi‐squared test evaluates the importance of each term in the model (Colony Choice ~ Established colony density + Alternative colony type), while the likelihood ratio test evaluates the fit of the whole model against a simple null model. *β* is the regression coefficient and provides an indication of effect size.

### 3.3 Does the presence of adults on a coral colony predict juvenile habitat use?

Juveniles were more likely to be present on a dead coral if there was an adult present, and the likelihood increased with the proportion of dead coral on a transect (Figure 4, linear regression, interaction *p* < 0.0001, Adj *R* ^2^ = 0.74, *F* = 138.4_(4183)_). This relationship was driven by colonies on transects with more than 50% dead coral, as *P. moluccensis* strongly avoided dead coral colonies on transects with lower levels of damage. However, on transects with higher levels of degradation, *P. moluccensis* selected dead corals with adults on them.

### 3.4 Effects of habitat loss on *P. moluccensis* abundance

There was a 54% decline in the abundance of adult *P. moluccensis* between the first and second survey time (from 0.39 *P. moluccensis* m^−1^ ± 0.05 SEM to 0.18 ± 0.02; *F* = 13.45_(1,87)_, *p* < 0.001). In contrast, juvenile abundance showed no significant change over the same period (17% decline, from 0.06 ± 0.01 to 0.04 ± 0.01; *F* = 0.7_(1,87)_, *p* = 0.4).

## 4 DISCUSSION

Here, we demonstrate a potential double jeopardy effect caused by density‐dependent habitat selection and a subsequent bandwagon effect due to conspecific attraction. Habitat use of adult *P. moluccensis* was dependent on the density of conspecifics on remnant live coral colonies and the proportion of dead coral colonies in the immediate surroundings. When around half of the corals in their immediate surroundings (i.e. transect level) are dead, adult fish start occurring on previously unutilised dead coral habitat, while remnant live corals are crowded with high densities of *P. moluccensis*. In our habitat choice experiment, we confirmed this density‐dependent habitat selection behaviour, as fish increasingly selected an alternate colony as the established colony becomes more crowded. However, at lower densities, they consistently chose the live coral colony over a dead one. Juvenile *P. moluccensis* were unlikely to select dead coral habitat on the reef unless it had a prior resident, suggesting conspecific attraction is more important than other habitat quality cues despite available live coral habitat nearby. We argue that this change in habitat use, initiated by density‐dependent selection in adults, creates a bandwagon effect where the next generation selects dead corals over live due to conspecific attraction.

The habitat use of *P. moluccensis* adults was affected by the proportion of dead coral on each transect. On reefs where the damage from crown‐of‐thorns starfish was low, *P. moluccensis* adults almost exclusively used live coral. In contrast, adults started occupying dead coral colonies when the surrounding reef contained a high proportion of dead coral per transect. Under natural conditions, these live coral specialists preferentially select branching and corymbose Acropora colonies when they recruit to the reef (Bonin, 2012). Furthermore, when faced with the choice between a live and a dead coral in an experimental setting, *P. moluccensis* consistently chooses live coral (Feary et al., 2007; Öhman et al., 1998) and vacates dead coral colonies in search of live habitat nearby (Coker et al., 2012). Taken together, these studies support the interpretation that *P. moluccensis* can assess habitat quality and may suggest that the observed occupancy of dead coral in our study reflects behavioural flexibility in response to crowding. The shift in habitat use to dead coral colonies on highly disturbed reefs is most likely the result of density‐dependent habitat selection, triggered by displaced individuals from dead colonies crowding onto remnant live colonies. Thus, the movement of adults may be dictated by a combination of the degree of coral loss in the immediate vicinity and the availability of alternate habitat locally. It should be noted that this interpretation is based on static spatial data, and we acknowledge that other processes such as differential survival could also contribute to the observed pattern. However, this kind of spatial mismatch between live coral loss and the local abundance of *P. moluccensis* adults was described on the Great Barrier Reef following the large‐scale bleaching event of 2016 (Wismer et al., 2019). Furthermore, the core movement areas of *P. moluccensis* individuals tend to increase with body size and as live coral declines, suggesting a degree of flexibility in habitat use in response to disturbance events (Streit et al., 2021).

Relocation to remnant live habitat following disturbance events is well documented in mobile species. For example, oystercatchers have been shown to abandon disturbed feeding grounds in favour of intact patches (Goss‐Custard, 1977; Goss‐Custard et al., 2006). In our study, the average density of *P. moluccensis* per live coral colony more than doubled on reefs with >75% dead coral compared to those with <25%. Such crowding is likely to intensify competition and aggression (Bonin et al., 2015; Boström‐Einarsson et al., 2014; Ward et al., 2006), which in turn can lead to both lethal (Brunton & Booth, 2003) and sublethal (Booth, 1995) effects. Under these conditions, relocating to less crowded but lower quality habitat may improve individual fitness, as predicted by the ideal free distribution (Fretwell & Lucas, 1969). While density‐dependent habitat selection has been demonstrated in many taxa and systems including salmon (Falcy, 2015), ducks (Harper, 1982), forest birds (Krebs, 1971), freshwater fish (Milinski, 2010) and sheep (Mobaek et al., 2009), this study is among the first to show how habitat degradation can trigger such dynamics in a coral reef fish.

Our behavioural experiment demonstrated that *P. moluccensis* engages in density‐dependent habitat selection and confirms the relative roles of conspecific attraction versus resource quality. When choosing between a coral colony with an established group of conspecifics and an alternate colony with a pair of conspecifics, juvenile *P. moluccensis* consistently chose the established coral colony, as long as the densities remained relatively low. However, when densities increased above those regularly observed on undisturbed reefs (>317 m^−2^), the juvenile was more likely to choose the alternate colony. Furthermore, the point where a majority of individuals chose the alternate colony did not differ significantly between live and dead coral, suggesting that crowding on remnant live colonies may create conditions of perceived or actual resource limitation, consistent with previous studies on density‐dependent mortality and aggression in this species (Booth, 1995; Brunton & Booth, 2003).

Juvenile *P. moluccensis* were more likely to select dead coral habitat when adults were already present, despite the general avoidance of dead coral on less degraded reefs. Although our surveys do not directly capture the moment of settlement, previous work on *P. moluccensis* (Öhman et al., 1998) has shown that naive larvae and newly settled juveniles exhibit similar and persistent habitat preferences, specifically, a preference for live coral and for habitats occupied by conspecifics. This suggests that juvenile presence on particular colony types, especially in association with adult residents, can serve as a meaningful proxy for settlement preference. We propose that this shift in habitat preference reflects a socially driven feedback loop, a bandwagon effect, in which juveniles use adult presence as a settlement cue, regardless of habitat quality. The bandwagon effect captures the idea of behavioural conformity cascading across generations, driven by social information rather than direct assessment of habitat quality.

While these findings are consistent with the broad predictions of density‐dependent habitat selection, they differ mechanistically from classical models such as Shima and Osenberg's cryptic density‐dependent framework (Osenberg et al., 2006; Shima & Osenberg, 2003). In their model, settlement decisions are shaped by an integrated assessment of habitat quality and local conspecific density, made by individuals at the time of settlement. In contrast, our study describes a two‐step process: First, adult fish shift their distribution in response to crowding on remnant live coral; second, juvenile settlement is subsequently influenced by the presence of those adults, even on degraded habitats. This socially mediated cue overrides direct habitat assessment, leading to settlement decisions that may not optimise fitness, a pattern more consistent with a perceptual trap or bandwagon effect. Furthermore, this pattern shares clear conceptual similarities with ecological traps, particularly those driven by perceptual errors where formerly reliable cues (e.g. conspecific presence) no longer indicate high‐quality habitat (Robertson & Hutto, 2006; Hale & Swearer, 2016; Schlaepfer et al., 2002). Indeed, perceptual traps have been described in a range of systems, such as birds using social cues to select nesting sites (e.g. Nocera et al., 2005; Serrano & Tella, 2007), insects responding maladaptively to visual or chemical cues (Kriska, 1998) and fish aggregating in overfished zones due to degraded, yet socially populated, habitat patches (Battin, 2004). Our case shares the perceptual mechanism (reliance on conspecific cues) but differs in its transient nature and the timing of the response. The bandwagon effect appears to arise rapidly during or immediately following habitat loss, when adult fish are temporarily displaced onto dead coral due to crowding. Over time, these individuals may disperse, relocate to better habitat or experience sublethal effects that diminish their viability as settlement cues. This likely explains why such patterns may not persist or be observed in longer term surveys of post‐disturbance systems. Our findings therefore highlight a short‐lived, socially mediated feedback that can amplify the immediate behavioural consequences of coral loss. Furthermore, as the concept of an ecological trap carries a strict definition, typically requiring evidence of a preference for low‐quality habitat and a direct reduction in fitness, we believe it is important to apply the term cautiously.

Our study satisfies some, but not all, of the criteria for demonstrating an ecological trap. We show that juveniles increasingly select degraded (dead coral) habitat in the presence of adults, despite available live coral alternatives, and that this behaviour becomes more common on highly disturbed reefs. Prior work also supports the assumption that dead coral is associated with lower survival and reduced anti‐predator behaviour in this species (e.g. Bonin et al., 2009; Boström‐Einarsson et al., 2018). Indeed, this study recorded a 54% reduction in the abundance of *P. moluccensis* adults in the 12 months following the start of the crown‐of‐thorns outbreak. The observed decline in adult abundance is consistent with prior findings of reduced survival in degraded habitats (e.g. Boström‐Einarsson et al., 2018), though we acknowledge that other processes, such as movement or lower detection probability, may also have contributed. Interestingly, juvenile abundance did not change significantly over the same period. This is somewhat surprising given our spatial results, which showed shifts in habitat use and reduced occupancy of degraded habitat. One possible explanation is continued larval input into the system or the persistence of conspecific‐driven settlement behaviours irrespective of local adult density. However, these mechanisms remain speculative, and we did not directly measure fitness outcomes or demographic consequences in this study. We, therefore, suggest that the observed bandwagon effect represents a plausible mechanism by which ecological traps could emerge in this system, particularly if social cues continue to guide settlement into degraded habitat, and if such settlement leads to reduced fitness. Further work measuring survival and reproductive output of individuals on different coral types would be required to confirm this.

As coral reefs and global ecosystems are rapidly transforming into highly altered versions of their historical counterparts, ecological traps may emerge as an unexpected consequence of environmental change. Here, we describe how juveniles settle on dead coral habitat that they would otherwise rarely select, attracted by the presence of adult conspecifics. This type of socially mediated habitat choice has been predicted to lead to extinction of populations in simulations linking ecological traps and conspecific attraction (Swartwout, 2007), yet has not previously been demonstrated. These results provide insight into how habitat loss interacts with density‐dependent habitat selection to potentially create a perceptual trap with consequences at the population level. These findings also highlight the critical importance of field‐collected data, which allow the discovery of secondary consequences of environmental change that may be overlooked in more controlled experimental settings. Given the accelerating pace of ecosystem change and the increasing decoupling of historical cues from habitat quality, it is critical for conservation and management efforts to consider the potential for socially mediated ecological traps and their role in shaping species persistence in novel environments.

## AUTHOR CONTRIBUTIONS

Lisa Boström Einarsson conceived the ideas and designed methodology, collected and analysed the data, with substantial input and feedback from Mary C. Bonin, Geoffrey P. Jones and Philip L. Munday. Sally A. Keith contributed to the development of the conceptual framework and integration with ecological theory. Lisa Boström Einarsson led the writing of the manuscript. All authors contributed critically to the drafts and gave final approval for publication.

## CONFLICT OF INTEREST STATEMENT

The authors declare no conflict of interest.

## Supporting information


**Figure S1.** Change point analysis of the density of *P. moluccensis* modelled against the proportion dead coral on each transect (Figure 2a). Model tested includes a sigma variance parameter testing for a variance change point. Grey fitted lines are drawn randomly from the posterior (9000 iterations), the blue change point represents the posterior density for each chain (*n* = 3) and the green dashed line indicates the variance prediction intervals.

## Data Availability

Data available from the Dryad Digital Repository https://doi.org/10.5061/dryad.8w9ghx40z (Boström‐Einarsson et al., 2025).

## References

[jane70135-bib-0001] Atema, J. , Kingsford, M. J. , & Gerlach, G. (2002). Larval reef fish could use odour for detection, retention and orientation to reefs. Marine Ecology Progress Series, 241, 151–160.

[jane70135-bib-0002] Battin, J. (2004). When good animals love bad habitats: Ecological traps and the conservation of animal populations. Conservation Biology, 18(6), 1482–1491.

[jane70135-bib-0005] Bonin, M. C. (2012). Specializing on vulnerable habitat: Acropora selectivity among damselfish recruits and the risk of bleaching‐induced habitat loss. Coral Reefs, 31(1), 287–297.

[jane70135-bib-0006] Bonin, M. C. , Boström‐Einarsson, L. , Munday, P. L. , & Jones, G. P. (2015). The prevalence and importance of competition among coral reef fishes. Annual Review of Ecology, Evolution, and Systematics, 46(1), 169–190.

[jane70135-bib-0007] Bonin, M. C. , Munday, P. L. , McCormick, M. I. , Srinivasan, M. , & Jones, G. P. (2009). Coral‐dwelling fishes resistant to bleaching but not to mortality of host corals. Marine Ecology Progress Series, 394, 215–222.

[jane70135-bib-0008] Booth, D. J. (1995). Juvenile groups in a coral‐reef damselfish: Density‐dependent effects on individual fitness and population demography. Ecology, 76(1), 91–106.

[jane70135-bib-0009] Booth, D. J. , & Beretta, G. A. (2004). Influence of recruit condition on food competition and predation risk in a coral reef fish. Oecologia, 140(2), 289–294. 15179583 10.1007/s00442-004-1608-1

[jane70135-bib-0010] Boström‐Einarsson, L. , Bonin, M. C. , & Munday, P. L. (2014). Habitat degradation modifies the strength of interspecific competition in coral dwelling damselfishes. Ecology, 95, 3056–3067. https://esajournals.onlinelibrary.wiley.com/doi/abs/10.1890/13‐1345.1

[jane70135-bib-0011] Boström‐Einarsson, L. , Bonin, M. C. , Munday, P. L. , & Jones, G. P. (2018). Loss of live coral compromises predator‐avoidance behaviour in coral reef damselfish. Scientific Reports, 8(1), 7795. 29773843 10.1038/s41598-018-26090-4 PMC5958076

[jane70135-bib-0012] Boström‐Einarsson, L. , Bonin, M. C. , Munday, P. L. , Jones, G. P. , & Keith, S. A. (2025). Data from: Density dependent habitat selection in response to habitat loss in a coral reef fish. *Dryad Digital Repository*. 10.5061/dryad.8w9ghx40z PMC12673249 40974053

[jane70135-bib-0013] Boto‐García, D. , & Baños‐Pino, J. F. (2022). Social influence and bandwagon effects in tourism travel. Annals of Tourism Research, 93, 103366.

[jane70135-bib-0014] Brooker, R. M. , Jones, G. P. , & Munday, P. L. (2013). Prey selectivity affects reproductive success of a corallivorous reef fish. Oecologia, 172(2), 409–416. 23124333 10.1007/s00442-012-2521-7

[jane70135-bib-0015] Brunton, B. J. , & Booth, D. J. (2003). Density‐ and size‐dependent mortality of a settling coral‐reef damselfish (*Pomacentrus moluccensis* Bleeker). Oecologia, 137(3), 377–384. 13680350 10.1007/s00442-003-1377-2

[jane70135-bib-0016] Buxton, V. L. , Enos, J. K. , Sperry, J. H. , & Ward, M. P. (2020). A review of conspecific attraction for habitat selection across taxa. Ecology and Evolution, 10(23), 12690–12699. 33304487 10.1002/ece3.6922 PMC7713925

[jane70135-bib-0017] Calenge, C. (2006). The package “adehabitat” for the R software: A tool for the analysis of space and habitat use by animals. Ecological Modelling, 197, 516–519.

[jane70135-bib-0018] Coker, D. J. , Pratchett, M. S. , & Munday, P. L. (2012). Influence of coral bleaching, coral mortality and conspecific aggression on movement and distribution of coral‐dwelling fish. Journal of Experimental Marine Biology and Ecology, 414‐415, 62–68.

[jane70135-bib-0019] Coker, D. J. , Walker, S. P. W. , Munday, P. L. , & Pratchett, M. S. (2013). Social group entry rules may limit population resilience to patchy habitat disturbance. Marine Ecology Progress Series, 493, 237–242.

[jane70135-bib-0020] Cole, A. J. , Pratchett, M. S. , & Jones, G. P. (2008). Diversity and functional importance of coral‐feeding fishes on tropical coral reefs. Fish and Fisheries, 9, 286–307. https://onlinelibrary.wiley.com/doi/abs/10.1111/j.1467‐2979.2008.00290.x

[jane70135-bib-0021] Damgaard, C. (2019). A critique of the space‐for‐time substitution practice in community ecology. Trends in Ecology & Evolution, 34(5), 416–421. 30824195 10.1016/j.tree.2019.01.013

[jane70135-bib-0022] Falcy, M. R. (2015). Density‐dependent habitat selection of spawning Chinook salmon: Broad‐scale evidence and implications. The Journal of Animal Ecology, 84(2), 545–553. 25283166 10.1111/1365-2656.12297

[jane70135-bib-0023] Farjam, M. (2021). The bandwagon effect in an online voting experiment with real political organizations. International Journal of Public Opinion Research, 33(2), 412–421.

[jane70135-bib-0024] Feary, D. A. , Almany, G. R. , McCormick, M. I. , & Jones, G. P. (2007). Habitat choice, recruitment and the response of coral reef fishes to coral degradation. Oecologia, 153(3), 727–737. 17566781 10.1007/s00442-007-0773-4

[jane70135-bib-0025] Feary, D. A. , McCormick, M. I. , & Jones, G. P. (2009). Growth of reef fishes in response to live coral cover. Journal of Experimental Marine Biology and Ecology, 373(1), 45–49.

[jane70135-bib-0027] Forrester, G. E. , Evans, B. , Steele, M. A. , & Vance, R. R. (2006). Assessing the magnitude of intra‐ and interspecific competition in two coral reef fishes. Oecologia, 148(4), 632–640. 16518631 10.1007/s00442-006-0397-0

[jane70135-bib-0028] Fretwell, S. D. (1972). Populations in a seasonal environment (MPB‐5). Princeton University Press. 4680650

[jane70135-bib-0029] Fretwell, S. D. , & Lucas, H. L. (1969). On territorial behavior and other factors influencing habitat distribution in birds. Acta Biotheoretica, 19(1), 16–36.

[jane70135-bib-0030] Goss‐Custard, J. D. (1977). The ecology of the Wash. III. Density‐related behaviour and the possible effects of a loss of feeding grounds on wading birds (Charadrii). The Journal of Applied Ecology, 14(3), 721–739.

[jane70135-bib-0031] Goss‐Custard, J. D. , Burton, N. H. K. , Clark, N. A. , Ferns, P. N. , McGrorty, S. , Reading, C. J. , Rehfisch, M. M. , Stillman, R. A. , Townend, I. , West, A. D. , & Worrall, D. H. (2006). Test of a behavior‐based individual‐based model: Response of shorebird mortality to habitat loss. Ecological Applications: A Publication of the Ecological Society of America, 16(6), 2215–2222. 17205899 10.1890/1051-0761(2006)016[2215:toabim]2.0.co;2

[jane70135-bib-0033] Hale, R. , & Swearer, S. E. (2016). Ecological traps: Current evidence and future directions. Proceedings of the Royal Society B: Biological Sciences, 283(1824), 20152647. 10.1098/rspb.2015.2647 PMC4760169 26865295

[jane70135-bib-0035] Harper, D. G. C. (1982). Competitive foraging in mallards: ‘ideal free’ ducks. Animal Behaviour, 30, 575–584. https://www.sciencedirect.com/science/article/pii/S0003347282800717

[jane70135-bib-0039] Hoey, A. S. , & McCormick, M. I. (2004). Selective predation for low body condition at the larval‐juvenile transition of a coral reef fish. Oecologia, 139(1), 23–29. 14767752 10.1007/s00442-004-1489-3

[jane70135-bib-0040] Holbrook, S. J. , & Schmitt, R. J. (2002). Competition for shelter space causes density‐dependent predation mortality in damselfishes. Ecology, 83(10), 2855–2868.

[jane70135-bib-0041] Hughes, T. P. , Kerry, J. T. , Connolly, S. R. , Álvarez‐Romero, J. G. , Eakin, C. M. , Heron, S. F. , Gonzalez, M. A. , & Moneghetti, J. (2021). Emergent properties in the responses of tropical corals to recurrent climate extremes. Current Biology: CB, 31, 5393–5399.e3. 10.1016/j.cub.2021.10.046 34739821

[jane70135-bib-0042] Inouye, B. D. (2001). Response surface experimental designs for investigating interspecific competition. Ecology, 82, 2696–2706. https://esajournals.onlinelibrary.wiley.com/doi/abs/10.1890/0012‐9658(2001)082[2696:RSEDFI]2.0.CO;2

[jane70135-bib-0043] Jones, G. P. , McCormick, M. I. , Srinivasan, M. , & Eagle, J. V. (2004). Coral decline threatens fish biodiversity in marine reserves. Proceedings of the National Academy of Sciences of the United States of America, 101(21), 8251–8253. 15150414 10.1073/pnas.0401277101 PMC419589

[jane70135-bib-0044] Kokita, T. , & Nakazono, A. (2001). Rapid response of an obligately corallivorous filefish *Oxymonacanthus longirostris* (Monacanthidae) to a mass coral bleaching event. Coral Reefs, 20(2), 155–158.

[jane70135-bib-0045] Krebs, J. R. (1971). Territory and breeding density in the great tit, *Parus major* L. Ecology, 52(1), 2–22.

[jane70135-bib-4000] Kriska, G. , Horváth, G. , & Andrikovics, S. (1998). Why Do Mayflies Lay Their Eggs En Masse on Dry Asphalt Roads? Water‐Imitating Polarized Light Reflected From Asphalt Attracts Ephemeroptera. Journal of Experimental Biology, 201(15), 2273–2286. 10.1242/jeb.201.15.2273 9662498

[jane70135-bib-0046] Lecchini, D. , Shima, J. , Banaigs, B. , & Galzin, R. (2005). Larval sensory abilities and mechanisms of habitat selection of a coral reef fish during settlement. Oecologia, 143(2), 326–334. 15647903 10.1007/s00442-004-1805-y

[jane70135-bib-0047] Leibenstein, H. (1950). Bandwagon, snob, and Veblen effects in the theory of consumers' demand. The Quarterly Journal of Economics, 64(2), 183–207.

[jane70135-bib-0048] Lindeløv, J. K. (2020). *mcp*: *An R package for regression with multiple change points* . 10.31219/osf.io/fzqxv

[jane70135-bib-0049] Macarthur, R. , & Levins, R. (1964). Competition, habitat selection, and character displacement in a patchy environment. Proceedings of the National Academy of Sciences of the United States of America, 51, 1207–1210. 14215645 10.1073/pnas.51.6.1207 PMC300237

[jane70135-bib-0050] Majoris, J. E. , Foretich, M. A. , Hu, Y. , Nickles, K. R. , Di Persia, C. L. , Chaput, R. , Schlatter, E. , Webb, J. F. , Paris, C. B. , & Buston, P. M. (2021). An integrative investigation of sensory organ development and orientation behavior throughout the larval phase of a coral reef fish. Scientific Reports, 11(1), 12377. 34117298 10.1038/s41598-021-91640-2 PMC8196062

[jane70135-bib-0052] Milinski, M. (2010). An evolutionarily stable feeding strategy in Sticklebacks1. Zeitschrift für Tierpsychologie, 51(1), 36–40.

[jane70135-bib-0053] Mobaek, R. , Mysterud, A. , Egil Loe, L. , Holand, Ø. , & Austrheim, G. (2009). Density dependent and temporal variability in habitat selection by a large herbivore; an experimental approach. Oikos, 118(2), 209–218.

[jane70135-bib-0054] Morris, D. W. (1989). Density‐dependent habitat selection: Testing the theory with fitness data. Evolutionary Ecology, 3(1), 80–94.

[jane70135-bib-0055] Nadeau, R. , Cloutier, E. , & Guay, J.‐H. (1993). New evidence about the existence of a bandwagon effect in the opinion formation process. International Political Science Review, 14(2), 203–213.

[jane70135-bib-3000] Nocera, J. J. , Forbes, G. J. , & Giraldeau, L.‐A. (2005). Inadvertent Social Information in Breeding Site Selection of Natal Dispersing Birds. Proceedings of the Royal Society B: Biological Sciences, 273(1584), 349–355. 10.1098/rspb.2005.3318 PMC1560037 16543178

[jane70135-bib-0056] Noonan, S. H. C. , Jones, G. P. , & Pratchett, M. S. (2012). Coral size, health and structural complexity: Effects on the ecology of a coral reef damselfish. Marine Ecology Progress Series, 456, 127–137.

[jane70135-bib-0057] Öhman, M. C. , Munday, P. L. , Jones, G. P. , & Caley, M. J. (1998). Settlement strategies and distribution patterns of coral‐reef fishes. Journal of Experimental Marine Biology and Ecology, 225(2), 219–238.

[jane70135-bib-0058] Osenberg, C. W. , Shima, J. S. , & St Mary, C. M. (2006). Habitat degradation and settlement behavior: Effects on fish settlement, survival, and recruitment. In Proceedings of the 10th International Coral Reef Symposium, Okinawa, Japan. Japanese Coral Reef Society, Tokyo, Japan (pp. 257–263). Japanese Coral Society.

[jane70135-bib-0059] Pickett, S. T. A. (1989). Space‐for‐time substitution as an alternative to long‐term studies in ecology. In G. E. Likens (Ed.), Long‐term studies in ecology: Approaches and alternatives (pp. 110–135). Springer‐Verlag.

[jane70135-bib-0061] Pratchett, M. S. , Thompson, C. A. , Hoey, A. S. , Cowman, P. F. , & Wilson, S. K. (2018). Effects of coral bleaching and coral loss on the structure and function of reef fish assemblages. In M. J. H. van Oppen & J. M. Lough (Eds.), Coral bleaching: Patterns, processes, causes and consequences (pp. 265–293). Springer International Publishing.

[jane70135-bib-0062] Pratchett, M. S. , Wilson, S. K. , Berumen, M. L. , & McCormick, M. I. (2004). Sublethal effects of coral bleaching on an obligate coral feeding butterflyfish? Coral Reefs, 23(3), 352–356.

[jane70135-bib-0063] Pulliam, H. R. , & Danielson, B. J. (1991). Sources, sinks, and habitat selection: A landscape perspective on population dynamics. The American Naturalist, 137, S50–S66.

[jane70135-bib-0064] Robertson, B. A. , & Hutto, R. L. (2006). A framework for understanding ecological traps and an evaluation of existing evidence. Ecology, 87(5), 1075–1085. 16761584 10.1890/0012-9658(2006)87[1075:affuet]2.0.co;2

[jane70135-bib-0065] Rohlfs, J. H. (2003). Bandwagon effects in high‐technology industries. MIT Press.

[jane70135-bib-0066] Rosenzweig, M. L. (1981). A theory of habitat selection. Ecology, 62(2), 327–335.

[jane70135-bib-2000] Schlaepfer, M. A. , Runge, M. C. , & Sherman, P. W. (2002). Ecological and Evolutionary Traps. Trends in Ecology & Evolution, 17(10), 474–480. 10.1016/s0169-5347(02)02580-6

[jane70135-bib-1000] Schneider, C. A. , Rasband, W. S. , & Eliceiri, K. W. (2012). NIH Image to ImageJ: 25 Years of Image Analysis. Nature Methods, 9(7), 671–675. 10.1038/nmeth.2089 22930834 PMC5554542

[jane70135-bib-0069] Serrano, D. , & Tella, J. L. (2007). The role of despotism and heritability in determining settlement patterns in the colonial lesser kestrel. The American Naturalist, 169(2), E53–E67. 10.1086/510598 17211799

[jane70135-bib-0070] Shima, J. S. , & Osenberg, C. W. (2003). Cryptic density dependence: Effects of covariation between density and site quality in reef fish. Ecology, 84(1), 46–52.

[jane70135-bib-0071] Souter, D. , Planes, S. , Wicquart, J. , Logan, M. , Obura, D. , & Staub, F. (2020). Status of coral reefs of the world (Souter, D., Planes, S., Wicquart, J., Logan, M., Obura, D., & Staub, F. (Eds.)). ICRI, GCRMN, Australia Institute of Marine Science, UNEP.

[jane70135-bib-0072] Stamps, J. , & Krishnan, V. V. (2005). Nonintuitive cue use in habitat selection. Ecology, 86(11), 2860–2867.

[jane70135-bib-0073] Streit, R. P. , Hemingson, C. R. , Cumming, G. S. , & Bellwood, D. R. (2021). How flexible are habitat specialists? Short‐term space use in obligate coral‐dwelling damselfishes. Reviews in Fish Biology and Fisheries, 31, 381–398. 10.1007/s11160-021-09646-y

[jane70135-bib-0074] Swartwout, P. (2007). The bandwagon effect: Conspecific attraction and vulnerability to ecological traps. The Ohio State University. https://kb.osu.edu/handle/1811/28924

[jane70135-bib-0075] Sweatman, H. P. A. (1983). Influence of conspecifics on choice of settlement sites by larvae of two pomacentrid fishes (*Dascyllus aruanus* and *D. reticulatus*) on coral reefs. Marine Biology, 75(2), 225–229.

[jane70135-bib-0076] Sweet, M. , Burian, A. , & Bulling, M. (2021). Corals as canaries in the coalmine: Towards the incorporation of marine ecosystems into the ‘one health’ concept. Journal of Invertebrate Pathology, 186, 107538. https://www.sciencedirect.com/science/article/pii/S0022201121000057 33545133 10.1016/j.jip.2021.107538

[jane70135-bib-0078] Tolimieri, N. , Jeffs, A. , & Montgomery, J. C. (2000). Ambient sound as a cue for navigation by the pelagic larvae of reef fishes. Marine Ecology Progress Series, 207, 219–224.

[jane70135-bib-0079] Ward, A. J. W. , Kent, M. I. A. , & Webster, M. M. (2020). Social recognition and social attraction in group‐living fishes. Frontiers in Ecology and Evolution, 8, 15. 10.3389/fevo.2020.00015

[jane70135-bib-0080] Ward, A. J. W. , Webster, M. M. , & Hart, P. J. B. (2006). Intraspecific food competition in fishes. Fish and Fisheries, 7(4), 231–261.

[jane70135-bib-0082] Wismer, S. , Tebbett, S. B. , Streit, R. P. , & Bellwood, D. R. (2019). Spatial mismatch in fish and coral loss following 2016 mass coral bleaching. The Science of the Total Environment, 650(Pt 1), 1487–1498. 30308835 10.1016/j.scitotenv.2018.09.114

