# Supplementary material for: Density dependent habitat selection in response to habitat loss in a coral reef fish
Source: J Anim Ecol. 2025 Sep 19;94(12):2421–30. doi: 10.1111/1365-2656.70135 (PMC12673249; doi:10.1111/1365-2656.70135)
Supplement: Supplementary file 1 — Figure S1. Change point analysis of the density of P. moluccensis modelled against the proportion dead coral on each transect (Figure 2a). Model tested includes a sigma variance parameter testing for a variance change point. Grey fitted lines are drawn randomly from the posterior (9000 iterations), the blue change point represents the posterior density for each chain (n = 3) and the green dashed line indicates the variance prediction intervals. [file JANE-94-2421-s001.docx]

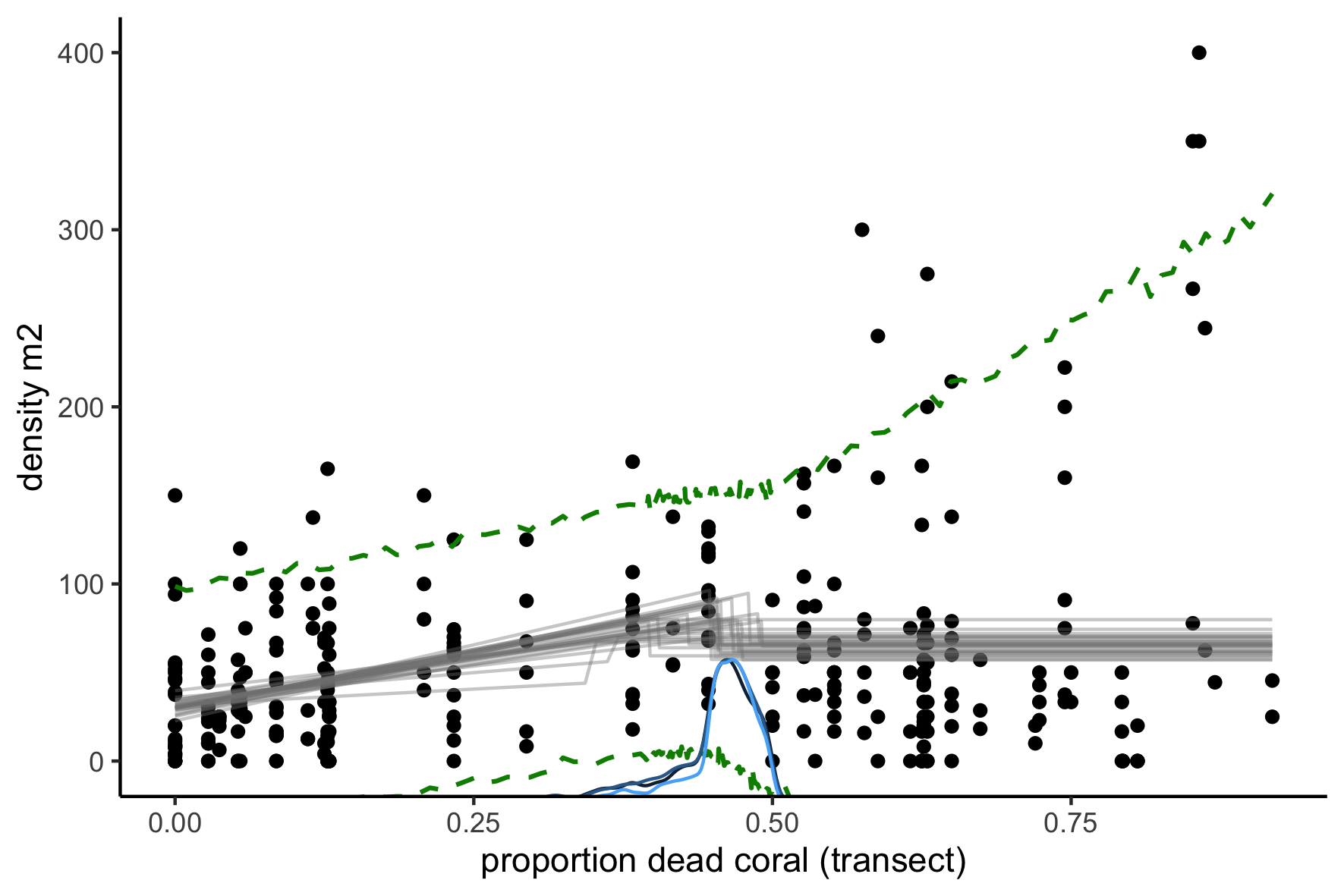


**Supplemental figure 1:** Change point analysis of the density of *P. moluccensis* modelled against the proportion dead coral on each transect (Figure 2a). Model tested includes a sigma variance parameter testing for a variance change point. Grey fitted lines are drawn randomly from the posterior (9000 iterations), the blue change point represents the posterior density for each chain (n=3), and the green dashed line indicates the variance prediction intervals.
